# The Utility of Animal Models for Studying the Metabo-Psychiatric Origins of Anorexia Nervosa

**DOI:** 10.3389/fpsyt.2021.711181

**Published:** 2021-10-14

**Authors:** Jie Zhang, Stephanie C. Dulawa

**Affiliations:** Department of Psychiatry, University of California, San Diego, San Diego, CA, United States

**Keywords:** anorexia nervosa, animal model, activity based anorexia, hypothalamus, dopaminergic pathway, gut microbiome, GWAS

## Abstract

Anorexia nervosa (AN) is a severe eating disorder that primarily affects young women and girls, and is characterized by abnormal restrictive feeding and a dangerously low body-mass index. AN has one of the highest mortality rates of any psychiatric disorder, and no approved pharmacological treatments exist. Current psychological and behavioral treatments are largely ineffective, and relapse is common. Relatively little basic research has examined biological mechanisms that underlie AN compared to other major neuropsychiatric disorders. A recent large-scale genome-wide association study (GWAS) revealed that the genetic architecture of AN has strong metabolic as well as psychiatric origins, suggesting that AN should be reconceptualized as a metabo-psychiatric disorder. Therefore, identifying the metabo-psychiatric mechanisms that contribute to AN may be essential for developing effective treatments. This review focuses on animal models for studying the metabo-psychiatric mechanisms that may contribute to AN, with a focus on the activity-based anorexia (ABA) paradigm. We also highlight recent work using modern circuit-dissecting neuroscience techniques to uncover metabolic mechanisms that regulate ABA, and encourage further work to ultimately identify novel treatment strategies for AN.

## Introduction

Anorexia nervosa (AN) is a complex and serious illness primarily characterized by a low body-mass index (BMI), fear of gaining weight, and body image disturbance. Patients with AN also frequently engage in compulsive exercise. AN predominantly affects women and girls, with clinical populations showing a 10:1 female-to-male ratio (1, 2). The onset of illness is typically during middle to late adolescence, and runs a disabling and chronic course with up to 4% in lifetime prevalence (3). AN has one of the highest mortality rates of any psychiatric disorder, with a weighted mortality rate of 5.1 deaths per 1,000 person-years from meta-analysis (4). Relapse is frequent in individuals with AN despite receiving treatment, with reported rates ranging between 9 and 52% (5). Certain cognitive traits have been associated with AN and precede onset of the illness, including cognitive rigidity, anxiety, and perfectionism (6). Strong genetic correlations have been identified between the heritability of AN and other psychiatric disorders, namely obsessive-compulsive disorder, major depressive disorder, and schizophrenia (1). Thus, elucidating biological mechanisms in AN may also shed light on the underpinnings of other related psychiatric conditions and traits.

Recent findings from genetic, neuroimaging, metabolic, and microbiome studies in humans have provided important leads for animal model studies investigating the metabo-psychiatric mechanisms underlying AN. For example, a recent GWAS combining data from the Anorexia Nervosa Genetics Initiative and the Eating Disorders Working Group identified significant genetic correlations of AN with metabolic traits and psychiatric disorders, suggesting that AN should be reconceptualized as a metabo-psychiatric disorder (1). Low BMI has traditionally been thought to result from core psychological features of AN, such as a drive for thinness. Yet, this view has failed to explain the extreme difficulty that AN patients face in the recovery and maintenance of a healthy BMI. These novel genetic data strongly suggest that metabolic traits contribute significantly to the development of AN (1, 7, 8) and should also be a target for treatment. Indeed, current treatments focusing only on nutritional restorations and psychological symptoms have been largely ineffective (5, 9). Furthermore, neuroimaging studies have identified alterations in the density of dopamine receptors in AN (10) which have subsequently been found to alter metabolism in preclinical studies (11). Future research efforts into the etiology and treatment of AN should target metabolic mechanisms gone awry in the disorder to develop novel treatment strategies.

Preclinical work using animal models to investigate biological mechanisms underlying core features of AN has not been prioritized for several reasons. One is the historical focus on sociocultural factors thought to contribute to eating disorders, which may make animal models appear unfeasible. Two, the mistaken perception that an animal model should recapitulate all aspects of a disorder may also discourage the development of animal models for studying aspects of AN (12). In fact, the current approach to developing animal models for studying neuropsychiatric disorders is to model only an aspect or a core feature of the disorder, and determine whether the model exhibits predictive validity (12). Developing a model with a more narrow use often leads to pragmatic advantages in the conduct of mechanistic studies, and can also increase the confidence in the cross-species validity of the model (12). A substantial increase in animal model work will be required to identify the metabo-psychiatric underpinnings of AN.

**Figure 1:**
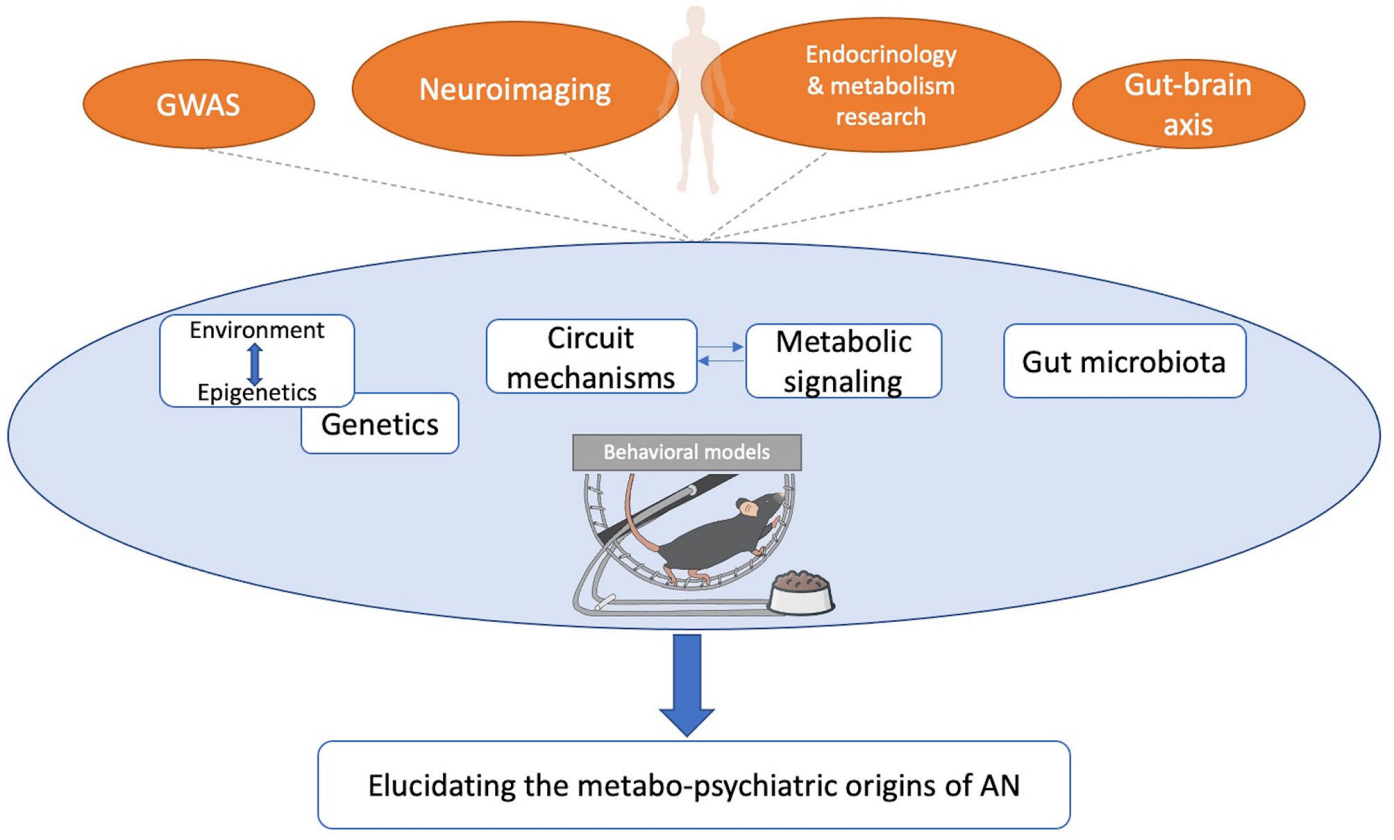
Framework for improving the utility of animal models of AN. Leads from human findings can be further tested and refined using animal models to elucidate the metabo-psychiatric origins of AN, and explore novel treatments. Findings from animal models can also generate testable hypotheses for human studies of AN. GWAS, genome-wide association study.

Our review highlights the utility of animal models for studying the metabo-psychiatric origins of AN. Recent work has combined the ABA paradigm with other modern techniques including circuit-dissecting approaches, genetic approaches, and gut microbiome manipulations. We review recent findings in this area, and encourage more preclinical work studying how metabolic mechanisms influence behaviors relevant to AN (Figure 1).

### Section 1. Overview of Activity-Based Anorexia

A commonly used biobehavioral animal model for aspects of AN is the activity-based anorexia (ABA) paradigm. In the ABA paradigm, rodents exposed to time-restricted feeding and constant running wheel access rapidly reduce food intake and bodyweight, and paradoxically develop hyperactivity (13). In contrast, rodents subjected to the same time-restricted feeding schedule without access to running wheels maintain body weight indefinitely. During ABA, rodents develop hypothermia (14, 15), loss of estrus, and increases in HPA axis activity (16, 17); if allowed to continue unchecked, ABA results in death (18). Importantly, the ABA phenomenon is highly conserved across mammalian species, and makes some accurate predictions about AN (18). For example, AN typically onsets during adolescence (6, 7), and younger rodents develop ABA more readily than older rodents (19, 20). Furthermore, female rats, and mice are more vulnerable to ABA than male rodents (18, 21), paralleling the female preponderance in AN.

The ABA paradigm recapitulates a core feature of AN, which is a paradoxical response to negative energy balance. In AN, individuals restrict feeding and engage in compulsive exercise while in a state of negative energy balance. When exposed to the ABA paradigm, rodents reduce voluntary food intake, and increase wheel running even as they progressively lose weight. The increase in wheel running in the ABA paradigm has been suggested to reflect increased foraging behavior (22). This hyperactivity often peaks before food delivery, and is termed food anticipatory activity (FAA), and has also been reported in AN patients (23). However, the ABA model does not recapitulate all aspects of AN. For example, providing high-fat food during the paradigm prevents the development of ABA (24). Furthermore, restoration of *ad-lib* feeding typically results in recovery of mice to a normal body weight (25), while AN patients do not readily recover only with presentation of food. Regardless, the ABA paradigm provides a useful model for a specific aspect of AN, which is the paradoxical response to negative energy balance under homeostatic feeding conditions.

Using the ABA model to identify metabolic mechanisms contributing to AN represents a tailored use of the model, which may lead to pragmatic advantages and aid in establishing cross-species validity (12). The ABA paradigm is ideally suited for assessing metabolic measures, and can be readily performed within metabolic chambers (26). The utility of the ABA paradigm for modelling cognitive aspects of AN is less well-established, although several reports lend support to this idea (27–29). Finally, the biological processes regulating feeding, activity, and metabolism are thought to be highly conserved between rodents and humans (30, 31), further supporting their use for studying metabolic mechanisms relevant to AN.

### Section 2. Use of the ABA Paradigm to Identify Metabo-Psychiatric Mechanisms in AN

**Figure 2:**
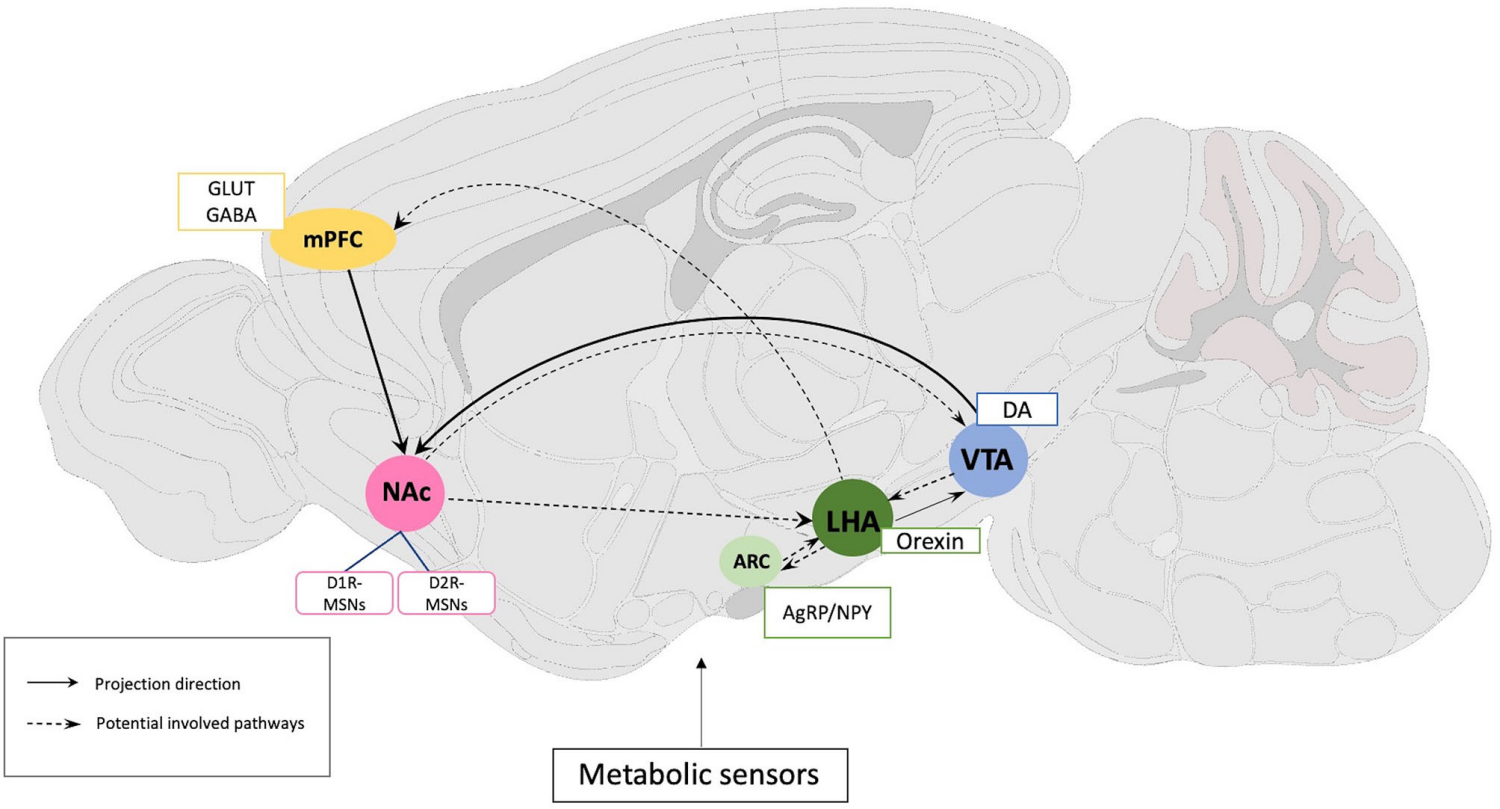
Schematic representation of rodent brain circuits and neural substrates implicated in the regulation of activity-based anorexia. Solid lines indicate established circuits, and dashed lines show potential circuits regulating the ABA phenomenon through effects on food intake, locomotor activity, FAA, or metabolic function. Primary neural substrates are listed in accordance with their brain regions. mPFC, Medial prefrontal cortex; NAc, Nucleus accumbens; ARC, Arcuate Nucleus; LHA, Lateral hypothalamus; VTA, Ventral tegmental area; NPY, Neuropeptide Y; AgRP, Agouti-related peptide; GLUT, glutamate; GABA, gamma-aminobutyric acid; DA, dopamine; D1R- and D2R-MSNs, dopamine D1 and D2 receptor expressing medium spiny neurons.

Most studies using the ABA paradigm have assessed only basic readouts of metabolic function including bodyweight, food intake, and wheel running activity during *ad libitum* vs. time-scheduled feeding. Far fewer studies have assessed metabolic measures more comprehensively. Modern circuit-dissecting approaches have identified some of the neural circuits that regulate metabolism in the context of ABA, although much work remains to be done in this area. Here, we highlight recent preclinical findings regarding the metabo-psychiatric mechanisms underlying ABA, including the role of the hypothalamic nuclei, striatal dopaminergic system, and gut-brain axis (Figure 2).

The hypothalamus is perhaps one of the most studied central brain regions which coordinates physiological and behavioral homeostasis and motivated behaviors including feeding and foraging (32, 33). It is thereby not surprising that altered hypothalamic structure and connectivity have been implicated in the emergence of AN. For example, neuroimaging studies have described increased connectivity within the arcuate nucleus (ARC) but decreased connectivity within the lateral hypothalamus, as well as neurochemical imbalances in AN patients following a meal (34–36). However, whether these and other structural and functional changes are a cause or consequence of AN remains unclear. Animal studies using the ABA paradigm have since assisted in probing the mechanisms by which hypothalamic dysregulation contributes to aspects of AN.

Agouti-related protein (AgRP)- and neuropeptide Y (NPY)-expressing neurons located within the ARC are activated by energy deficits and promote food-seeking behaviors and consumption (37). AN patients have been reported to have elevated plasma AgRP levels (38), and elevated concentrations of NPY in cerebrospinal fluid (39). In parallel, animals exhibit significantly higher AgRP/NPY mRNA levels in the ARC during ABA (40, 41), while intracerebroventricular infusion of NPY facilitates ABA by increasing running activity and decreasing food intake (42). Recent work using fiber photometry has provided novel insights into the role of AgRP neurons in regulating metabo-psychiatric processes underlying the ABA phenomenon. For example, Miletta et al. (24) demonstrated that ablation of AgRP neurons in the early postnatal period prevents fuel mobilization during ABA conditions, resulting in reduced running wheel activity and marked weight loss, whereas chemogenetic activation of AgRP neurons increases running wheel activity and extends survival in the paradigm. Furthermore, they also reported that AgRP neuron activity rapidly decreases with termination of running, revealing a novel role for AgRP neurons in regulating compulsive running behavior during ABA. Duriez et al. (40) reported complex effects of food restriction and running wheel availability on mRNA levels of AgRP and NPY. Specifically, they reported that mRNA levels of AgRP and NPY are increased during short- and long-term food restriction (2 vs. 10 weeks, respectively), but that the presence of a running wheel attenuates this increase. Further studies using modern circuit-dissecting approaches should clarify the complex role of AgRP and NPY in regulating metabo-psychiatric mechanisms of ABA.

Located exclusively in the lateral hypothalamus (LHA), orexin is another key neuropeptide thought to modulate reward, feeding, and activity (43). Orexin neurons project widely throughout the brain, and densely innervate the ARC (44). Plasma orexin-A levels have been reported to be elevated in untreated AN patients in some studies (45), and reduced in others (46). Orexin neuronal activity is rapidly inhibited following food consumption, while ablation of orexin neurons promotes overeating and obesity (47). Orexin neuron activity is increased during FAA (48, 49), and elevated spontaneous physical activity (SPA), which refers to physical activity not motivated by a reward. Furthermore, orexin neuron-ablated mice do not exhibit FAA when exposed to ABA conditions (49). Higher SPA levels have been reported to predict greater weight loss during ABA (50). Furthermore, when AgRP and orexin levels fail to upregulate during ABA in a rat model of passive stress coping, these rats lose weight more rapidly (51). More work will be required to clarify the role of orexin neuron activity in regulating ABA.

A large body of literature has implicated the striatal dopaminergic system in the etiology of AN. Much of this evidence comes from human neuroimaging studies revealing altered striatal function during tasks assessing reward, altered dopamine (DA) receptor levels, or altered dopamine metabolites (10, 52, 53). Recently, a small number of preclinical research studies have implicated the striatal dopaminergic system in metabolic processes affecting ABA. For example, chemogenetic excitation of the ventral tegmental area (VTA) to nucleus accumbens (NAc) pathway in female rats prevents weight loss during ABA by increasing food intake and FAA, without altering overall activity (54). Furthermore, hyperdopaminergia resulting from dopamine transporter knockdown accelerates the progression of ABA (55). Recently, viral overexpression of the D2 receptor expressing on the medium spiny neurons (D2-MSNs) of the nucleus accumbens core (D2R-OE_NAc_) was reported to induce rapid and robust weight loss in female, but not male, mice during ABA under mild food-restriction conditions. Of note, this sexually-dimorphic effect was also observed without running wheel access, and without alterations in food intake. In addition, D2R-OE_NAc_ mice showed robust glucose intolerance in the intraperitoneal glucose tolerance test (11), confirming a previous report that D2Rs in the NAc regulate glucose metabolism (56). Furthermore, experiments using chemogenetic approaches have revealed a role for cortico-striatal projections from medial prefrontal cortex (mPFC) to NAc shell in regulating body weight and activity during ABA (28), and these projections are modulated by dopamine (57). In summary, these findings highlight the need for more research to better characterize the role of striatal dopaminergic dynamics in ABA.

Mesolimbic DA neurons also detect peripheral signals relaying information regarding appetite and energy intake, such as leptin and ghrelin. Altered circulating concentrations of the orexigenic hormone ghrelin and the anorexigenic leptin have been reported in AN patient (58, 59). Mice treated with ghrelin increased food intake during ABA, although bodyweight was not affected (60). Furthermore, plasma ghrelin levels have been positively correlated with FAA, which can be suppressed by a ghrelin receptor (GHS-R1A) antagonist (61). Increases in ghrelin and reductions in leptin levels are thought to drive hyperactivity by directly altering the activity of dopamine neurons in the VTA (22). Complex dysfunction of nutrient-sensing systems may play a role in AN and involve interactions with the dopamine system, which regulates behavioral responses to food cues and influences energy homeostasis.

### Section 3. Use of the ABA Paradigm to Study the Role of the Gut-Brain Axis in AN

The gut microbiota is a complex community of trillions of microorganisms residing in the gastrointestinal tract, affecting both physiological and psychological health (62). The gut-brain axis has drawn more attention in AN research recently, as a fast growing body of literature suggests that alterations in gut microbiota might influence features of AN, including host energy hemostasis, appetite and body weight regulation, gastrointestinal symptomatology, and neurobehavioral traits including anxiety (63, 64). AN patients have been reported to exhibit gut dysbiosis, with imbalances in multiple microbes (8, 63). Interestingly, gut dysbiosis may be persistent even after short-term weight restoration in AN (65), suggesting that disrupted equilibrium of gut microbiota might be a causative factor in AN. However, the cause-effect relationship between the gut microbiota and AN still remains unclear. Studies using animal models will be essential for expanding our understanding of how the gut-brain axis contributes to AN pathology, beyond correlative findings.

Recently, preclinical research has substantiated that exposure to ABA conditions can affect gut microbiota composition and diversity primarily through metabolic processes induced by food restriction and the resulting negative energy balance (66, 67). ABA conditions have also been reported to induce specific proteome adaptations in gut bacteria favoring ATP production in response to the energy-restricted state of the host (68). On the other hand, the mechanisms by which gut microbiota influence AN risk and trajectory have yet to be determined. One study explored this direction by transplanting fecal microbiota derived from AN patients vs. healthy controls into germ-free mice. Interestingly, gut microbiome derived from AN patients significantly hindered the recipient mice from gaining body weight, and reduced in food intake and food efficiency ratio (body weight gain/food intake). Moreover, mice receiving fecal microbiota from AN patients also exhibit elevated anxiety-like behaviors in open field and marble-burying tests compared to mice receiving fecal microbiota from controls (69). Another report, in contrast, found no difference in body weight and lean/fat mass between AN recipient mice and control recipient mice after 4 weeks of colonization (70). However, neither study measured bodyweight or food intake under food restriction conditions, and only assessed these measures during *ad-lib* feeding. Studies employing the same strategy but testing animal recipients in the ABA paradigm will be essential to better understand whether the AN-derived gut microbiota contributes to the development of AN-like phenotypes.

### Section 4. Other Animal Models for Studying Metabo-Psychiatric Traits in AN

AN has an estimated twin-based heritability ranging from 30–80%, suggesting a strong genetic component to the risk of disease development. A recent large-scale GWAS identified eight significant loci that were associated with AN, including *CADM1, FOXP1*, and *PTBP2*, which are all expressed in the hypothalamus and other brain regions (1). Yet, these loci still require fine mapping in order to confirm the involvement of these genes. Once the functional consequences of these loci have been determined, genetic mouse models can be produced that will be essential for identifying AN disease mechanisms. Furthermore, GWAS could also be performed in mice to identify loci which regulate ABA, and these could be compared with the recent GWAS findings in AN.

Prior to the recent GWAS findings in AN, several animal studies reported mutations which induce a phenotype similar to AN. Early work reported that the *anx/anx* mice model mimics aspects of AN, including suppressed appetite and food intake, emaciated appearance, and premature death (71). Notably, the spontaneous *anx* mutation identified in mice induces abnormalities within the hypothalamus, involving an aberrant AgRP/NPY system (64, 72). Additionally, *anx/anx* mice display decreases in hypothalamic activity accompanied by impaired glucose utilization and energy metabolism (73). However, it remains unknown which gene is responsible for the phenotype of *anx/anx* mice, or whether AN patients show variation within the responsible gene.

A recent study reported that brain-specific knockout of *SIRT1*, a metabolic regulator responding to stress and nutrient availability, protects mice from ABA, while overexpression promotes the development of ABA including weight loss, hyperactivity, and anxiety (74). Although *SIRT1* has not been identified as a risk gene for AN in large-scale human genetic studies, these findings are consistent with metabo-psychiatric underpinnings of AN.

Adverse social and developmental experiences have been identified as risk factors for AN, and several animal models, especially maternal separation-based protocols, have been generated to examine the links between environmental influence and AN-related phenotypes (9). For example, maternal separation combined with time-restricted feeding alters the expression of genes involved in lipid and energy metabolism (75). Intriguingly, post-weaning isolation rearing induces FAA only in female mice during ABA (76), and maternal separation was found to upregulate DA expressing cells in the VTA on ABA rats in a sex-dependent manner (77). Furthermore, prenatal stress was reported to accelerate the progression of weight loss during ABA in passive stress-coping rats due to impairments of AgRP and orexin gene upregulation. Passive stress-coping rats are also suggested to have innate impairments in leptin and ghrelin in responses to starvation, which may underlie decreased food intake and associated heightened body weight loss during ABA (51). Prenatal stress was also found to accelerate ABA progression through the hypomethylation of placental miR-340, a sexually dimorphic regulator of nutrient transporters (78). Thus, environmental factors can induce epigenetic changes leading to metabolic alterations in rodent models, and thus may also be involved in triggering AN symptomatology in humans. In addition to the genetic and environmental models of AN highlighted here, others have been systematically reviewed elsewhere (29).

## Conclusion and Future Perspective

In summary, animal models are undoubtedly critical for identifying the metabo-psychiatric basis of AN and accelerating the discovery of novel treatments. Limitations to animal models for investigating AN include their inability to mimic certain psychological factors of the disorder including body image disturbances and fear of gaining weight. However, these limitations do not negate their usefulness for identifying the metabo-psychiatric mechanisms underlying the disorder. In particular, the ABA paradigm remains a promising and useful experimental preparation, especially when used in combination with modern circuit-dissecting techniques. Future research should pursue how traditionally viewed distinct circuits for metabolic regulation vs. psychiatric phenotypes interact to produce AN-like phenotypes. In addition, the development of new genetic and epigenetic animal models based on recent GWAS findings should have far-reaching impact. Another essential feature of AN and the ABA phenomenon which requires further mechanistic study is the robust sex difference in which females show far greater vulnerability. Indeed, recent work has revealed striatal manipulations which result in robust sex differences in vulnerability to ABA (11). In conclusion, animal models will be essential for identifying metabo-psychiatric mechanisms underlying vulnerability to AN, and for identifying the role of newly discovered genetic variants and gene pathways in AN.

## Author Contributions

JZ and SD conceptualized the manuscript. JZ wrote the first draft of the paper and constructed the figures. SD thoroughly reviewed and revised the manuscript. All authors finalized the manuscript.

## Conflict of Interest

The authors declare that the research was conducted in the absence of any commercial or financial relationships that could be construed as a potential conflict of interest.

## Publisher's Note

All claims expressed in this article are solely those of the authors and do not necessarily represent those of their affiliated organizations, or those of the publisher, the editors and the reviewers. Any product that may be evaluated in this article, or claim that may be made by its manufacturer, is not guaranteed or endorsed by the publisher.

## Abbreviations

ABA: activity-based anorexia
AgRP: agouti-related protein
AN: anorexia nervosa
ARC: arcuate nucleus
BMI: body-mass index
DA: dopamine
D1R- and D2R-MSNs: dopamine D1 and D2 receptor expressing medium spiny neurons
GHS-R1A: growth hormone secretagogue receptor 1A
GWAS: genome-wide association study
FAA: food anticipatory activity
LHA: lateral hypothalamus
mPFC: medial prefrontal cortex
NPY: neuropeptide Y
NAc: nucleus accumbens
SPA: spontaneous physical activity
VTA: Ventral tegmental area.
